# Yeast culture repairs rumen epithelial injury by regulating microbial communities and metabolites in sheep

**DOI:** 10.3389/fmicb.2023.1305772

**Published:** 2023-12-01

**Authors:** Huihui Wang, Manchun Su, Chunhui Wang, Dengpan Li, Qiao Li, Zilong Liu, Xingcai Qi, Yi Wu, Yongju Zhao, Taotao Li, Youji Ma

**Affiliations:** ^1^College of Animal Science and Technology, Gansu Agricultural University, Lanzhou, China; ^2^Gansu Key Laboratory of Animal Generational Physiology and Reproductive Regulation, Lanzhou, China; ^3^School of Agriculture and Forestry Technology, Longnan Teachers College, Chengxian, China; ^4^College of Animal Science and Technology, Southwest University, Chongqing, China

**Keywords:** yeast culture, rumen papillae, microorganism, metabolite, Hu sheep

## Abstract

This study delves into the impact of yeast culture (YC) on rumen epithelial development, microbiota, and metabolome, with the aim of investigating YC’s mechanism in regulating rumen fermentation. Thirty male lambs of Hu sheep with similar age and body weight were selected and randomly divided into three groups with 10 lambs in each group. Lambs were fed a total mixed ration [TMR; rough: concentrate (R:C) ratio ≈ 30:70] to meet their nutritional needs. The experiment adopted completely randomized design (CRD). The control group (CON) was fed the basal diet with high concentrate, to which 20 g/d of YC was added in the low dose YC group (LYC) and 40 g/d of YC in the high dose YC group (HYC). The pretrial period was 14 days, and the experimental trial period was 60 days. At the end of a 60-day trial, ruminal epithelial tissues were collected for histomorphological analysis, and rumen microorganisms were analyzed by 16S rDNA sequencing and rumen metabolites by untargeted liquid chromatography-mass spectrometry (LC–MS) metabolomics techniques. The results showed that YC improved rumen papilla development and increased rumen papilla length (*p* < 0.05), while decreased cuticle thickness (*p* < 0.05). The 16S rDNA sequencing results showed that YC reduced the relative abundance of *Prevotella_1* (*p* < 0.05), while significantly increased the relative abundance of *Ruminococcaceae_UCG-005*, *uncultured_bacterium_f_Lachnospiraceae*, and *Ruminococcus_1* genus (*p* < 0.05). Metabolomics analysis showed that YC changed the abundance of metabolites related to amino acid metabolism, lipid metabolism and vitamin metabolism pathways in the rumen. In summary, YC might maintain rumen health under high-concentrate diet conditions by changing rumen microbiota structure and fermentation patterns, thereby affecting rumen metabolic profiles and repairing rumen epithelial injury.

## Introduction

Microorganisms play a crucial role in maintaining endoenvironmental homeostasis, facilitating metabolism, regulating intestinal health, activating immune activity, and contributing to neurodevelopment. Gastrointestinal tract harbors a diverse range of microorganisms, which interact in symbiotic and competitive relationships, forming an abundant and complex microbial community (Yang et al., 2022). The rumen is an essential digestive organ for ruminants and is also the principal site for microbial fermentation of volatile fatty acids (Yang et al., 2023). At present, to pursue higher fattening efficiency, high-energy grain feed is often used in the feeding management of meat ruminants. This feeding mode improves production performance while also posing a threat to rumen health. Rapidly fermentable carbohydrates will cause abnormal rumen fermentation and thus resulting in a persistently low rumen pH, which increases the occurrence of subacute rumen acidosis (SARA) in ruminants, bringing a series of risks to animal health (Silberberg et al., 2013).

Yeast is one of the most commonly used probiotics in ruminant nutrition studies to improve feed efficiency and prevent rumen acidosis by improving rumen fermentation. It has been shown to be effective in restoring the microbial balance of the gastrointestinal tract, especially when animals are in a state of digestive disturbance or stress (Schofield et al., 2018; Aswal et al., 2023; Wang et al., 2023). As an important single-cell protein for animal feed, *Saccharomyces cerevisiae* (*S. cerevisiae*) is the most widely used yeast species in animal production, because it is rich in amino acids, peptides, nucleotides, yeast polysaccharides and some unknown growth-promoting active factors (di Nicolantonio et al., 2023). Studies have shown that yeast products containing *S. cerevisiae* are used as a feed additive in ruminant nutrition to effectively improve feed efficiency and performance, moderate ruminal pH and digestibility, and prevent ruminal acidosis (Ogunade et al., 2019). Yeast culture (YC) is a class of biological products that contain live yeast cells and metabolic by-products (e.g., enzymes, vitamins, etc.) produced by *S. cerevisiae* and some live yeast during fermentation (Liu et al., 2022). YC, as a green additive, has been widely used in ruminant production because of its positive effect on improving the production performance of ruminants (Bass et al., 2019; Pang et al., 2022). Studies have shown that YC significantly improves the dry matter and crude fiber digestibility and increases milk production in buffaloes (Hansen et al., 2017). Studies on lambs have shown that adding YC to the diet can effectively increase the digestibility of neutral detergent fiber and improve the slaughter performance (Moghaddam et al., 2021). A meta-analysis showed that adding YC to the diet contribute to increasing average daily feed intake, dry matter intake (DMI) and feed-to-weight ratio in beef cattle, and that the promotion effect became more significant with longer feeding time (Pas et al., 2016). Notably, the effects of YC on ruminant growth performance is not consistent. For example, a comprehensive analysis of the effects of YC on meat ruminants showed that the growth promotion ranged from insignificant to a 20% increase in daily weight gain, with an average gain of 8.7% (Denev et al., 2007). The main reasons for this seem to be related to factors such as product type, animal condition and use of different raw materials. At present, the less clarity on the action mechanism of YC on ruminants also limits the use of YC. Previous studies have shown that adding YC to high concentrate diets improves rumen fermentation and microbiota in ruminants (Kumar et al., 1997), which is of great importance for ruminant production and rumen health.

Our previous studies have shown that YC can effectively improve the production performance, feed digestibility and rumen fermentation mode of sheep fed with high-concentrate feed, increase the concentration of butyric acid and total volatile fatty acids (TVFA), increase cellulase activity, and reduce the concentration of harmful metabolites such as lactic acid, lipopolysaccharide and histamine, and affect the abundance of major functional flora (Su et al., 2022). Thus, we speculated that YC might affect rumen development and fermentation patterns by improving rumen microorganisms and metabolites. Based on these results, we further explored the effects of YC on rumen epithelial development, overall microbiota and metabolome in sheep, with the aim of clarifying the mechanisms of YC effects on rumen function.

## Materials and methods

### Ethics statement

All experimental procedures involving animals were approved by the Animal Care Committee of Gansu Agricultural University (GSAU-AEW-2020-0057).

### Animals and experimental design

Thirty male Hu male lambs with an initial average body weight of 19.27 ± 0.45 kg (3 months old) were randomly divided into 3 groups (10 lambs/group; *n* = 10) for completely randomized design (CRD). These lambs were housed in well-ventilated barns with free access to water in a medium-sized Hu sheep breeding farm (Qingyang, Gansu, China). Lambs were fed a total mixed ration (TMR) consisting of corn silage and grain mixtures to meet their nutritional requirements. Dietary ingredients and chemical composition of the basal diet were presented in Supplementary Table S1 (rough: concentrate (R:C) ratio ≈ 30: 70, dry matter (DM) basis). YC was provided by Phibro Animal Health Corporation, United States, the nutritional active ingredients: crude protein (CP) ≥ 12.0%, crude fat (EE) ≥ 3.0%, crude fiber (*CF*) ≤ 5.0%, Ash ≤3.0%, water ≤10.0%, *S. cerevisiae* ≥ 2 × 10^8^ CFU/g. The pretrial period was 14 days, and the experimental trial period was 60 days. Lambs were fed daily using a basal diet supplemented with 0 g (CON group), 20 g (LYC group), and 40 g (HYC group) yeast product each per day. For each group, the YC were accurately weighted and pre-mixed with a small amount of crushed grain, and then a homogeneous mixture was added to the basal feed to ensure accurate dispersion throughout the feed. At the same time, the same volume of crushed grain was subjected to the CON group. All lambs were fed twice a day (8:00 and 17:00 h).

### Sample collection and processing

At the end of the feeding experiment, six lambs were randomly selected in each group. After fasting for 12 h, the rumen fluid was collected through an oral catheter. To avoid contamination, the rumen fluid collected at the beginning was discarded, and the rumen fluid collected later was divided into two separate tubes. Immediately freeze with liquid nitrogen and store at −80°C for the determination of microbial diversity and metabolites in rumen fluid. Subsequently, three lambs of similar weight per group were selected and transported within 30 min (including careful catching and loading) to a local slaughterhouse (Qingyang, Gansu, China). The lambs underwent a standardized protocol for slaughter. The procedure used in this research adhered to the guidelines set out by the Animal Care and Use Committee of Gansu Agricultural University. After slaughter, the abdominal cavity was opened to remove the rumen and intestinal tract as a whole, and a rumen epithelial tissue sample (approximately 2 × 2 cm^2^) was carefully cut from the left rumen rucksack (Xie et al., 2020), and then fixed in 4% paraformaldehyde solution for hematoxylin–eosin (H&E) staining.

### Ruminal epithelial parameters

The fixed rumen epithelial tissue was dehydrated in gradient alcohol, then cleared in xylene and finally embedded in paraffin blocks. After trimming, the embedded wax blocks were cooled on a −20°C freezing table, and then the cooled wax blocks were sectioned on a paraffin microtome at a thickness of 4 μm. The slices were floated on a spreader in 40°C warm water to flatten the tissue, picked up and baked at 60°C in the oven. After dewaxing, the paraffin sections were washed with distilled water and stained with hematoxylin–eosin (HE), and then sealed with neutral gum (Wang et al., 2021). Using Nikon Eclipse E100 microscope (Japan), NIKON DS-U3 (Japan) imaging system and Image-Pro Plus 5.1 image analysis system to observe and measure the thickness of rumen horny layer and muscle layer, length and width of rumen papilla. Five complete discontinuous slices were obtained for each wax block, and more than three complete visual fields were selected for each slice to be measured (Zhen et al., 2023).

### Microbial diversity analysis

Eighteen rumen fluid samples collected through an oral catheter were subjected to 16S rDNA high-throughput sequencing in Biomarker Technologies (Beijing, China). The sequencing process was summarized as follows. After the total DNA of each sample was extracted, specific primers with Barcode were synthesized for PCR amplification and product purification, quantification and normalization to form a sequencing library (SMRT Bell). The quality inspection of the library was carried out first, and the qualified library was sequenced by PacBio Sequel. The off-machine data was exported to CCS files through the analysis software (SMRT Link, version8.0), and the CCS sequences of all samples were identified through the barcode sequence (lima software, v1.7.0) to remove chimeras, and finally high-quality CCS sequences were obtained. Use USEARCH (version 10.0) to cluster sequences at the level of similarity of 97%, and filter OTUs by default using 0.005% of all sequences sequenced as a threshold. Next, the sequences were divided into OTUs by the DADA2 method in QIIME2 (version 2020.6). Then species annotation, diversity analysis and difference analysis were performed based on OTUs. The analysis of the sequencing data was carried out using the BMKCloud platform.^1^ Alpha diversity indices including species richness (ACE and Chao1 indices), and diversity (Simpson and Shannon indices) were calculated based on the OTUs using QIIME2 (version 2020.6)^2^ (Li et al., 2023). Meanwhile, beta diversity analysis was used to evaluate differences between samples in species complexity (Wang et al., 2020). Based on the distance matrix obtained from the beta diversity analysis, principal coordinates analysis (PCoA) analysis was performed on the abundance matrix based on Brary-Curtis distance using R software package to further demonstrate the differences in species diversity among samples (Zhang et al., 2020). Next, Linear discriminant analysis effect size (LEfSe) software^3^ was used to make a LEfSe cluster diagram and linear discriminant analysis (LDA) diagram (Lin et al., 2022). The sequencing data was uploaded to the BioProject database (BioProject ID: PRJNA1023752).

### Rumen fluid metabolic profiles

After pretreatment, the rumen fluid was detected by liquid chromatography-mass spectrometry (LC–MS). The chromatograph was Waters UPLC Acquity I-Class PLUS (Waters, United States), and the mass spectrometer was Waters UPLC Xevo G2-XS QTOF (Waters, United States). The chromatographic column is Acquity UPLC HSS T3 (100 mm × 2.1 mm, 1.8 μm, Waters, the United States), the mass spectrometry scanning range is selected as mass-to-charge ratio (m/z) 50–1,200. Metabolome analysis in the present study was carried out at BMKCloud platform (see text footnote 1). The specific process was as follows. The data were normalized by the method of total peak area normalization to diminish the influence of uncorrelated factors, i.e., each metabolite of each sample was divided by the total peak area of the sample. To evaluate metabolome data reproducibility, partial least squares discrimination analysis (PLS-DA) was performed by MetaboAnalyst (version 4.0) to predict the differences in metabolic profiling between different groups, and then the results were visualized by R package ggplot2 (version 3.3.2) (Liu et al., 2022). Differentially accumulated metabolites were screened out, and the screening results of differential metabolites were visualized using a volcano plot. A heatmap was derived by clustering differential metabolites based on R software to compare the differences in metabolites in different groups. The Kyoto Encyclopedia of Genes and Genomes (KEGG) database was used to find enriched metabolic signaling pathways involving differential metabolites between two groups. The sankey plot was used to show the differential metabolites of top10 and their enriched metabolic pathways.

### Association analysis of 16S rDNA sequencing and metabolomics

Association analyses were carried out as described below. Correlation analysis between rumen microorganisms and metabolites used the R language cor.test function to calculate the Person correlation coefficient of species and metabolites. Next, the heatmap package visualizes correlation graphs.

### Statistical analysis

First, for rumen epithelial parameter data, five well-oriented papillae for each section were identified, and their lengths, widths, stratum corneum thicknesses, and muscle layer thickness were counted. For statistical analysis, parameter data for histometric measurements, such as papillae length, widths, stratum corneum thickness, and muscle layer thickness, were pooled by all sections to obtain a mean value for each epithelial parameter. The rumen epithelial parameters were sorted out with Excel 2019, and SPSS 23.0 software (SPSS Inc., Chicago, IL, United States) was used statistical analyses. Statistical analyses of results were performed with a one-way analysis of variance (one-way ANOVA), followed by the Duncans method for multiple comparisons between groups. All data were presented as mean ± standard error. Differences were considered significant at *p* < 0.05. Then, for rumen 16S data, LDA coupled with effect size measurements LEfSe analysis was applied to search for statistically significant biomarkers (LDA > 4, *p* < 0.05) among different groups using LEfSe software. Next, for rumen metabolome data, differences in metabolites between different groups were selected by combination of the fold change (FC > 1.5), the Student’s *t*-test (*p* < 0.05), and the variable importance in the projection (VIP) threshold (VIP > 1.0) (Li et al., 2022). And significantly enriched pathways were identified with a hypergeometric test’s *p*-value (Pang et al., 2023). Finally, screened out microbial species and metabolites with |r| > 0.6 and *p* < 0.05 using the R language (R-3.1.1) Hmisc package for correlation analysis (Bi et al., 2021).

## Results

### Effects of YC on growth performance, rumen epithelium development, and fermentation parameters in sheep

**Figure 1:**
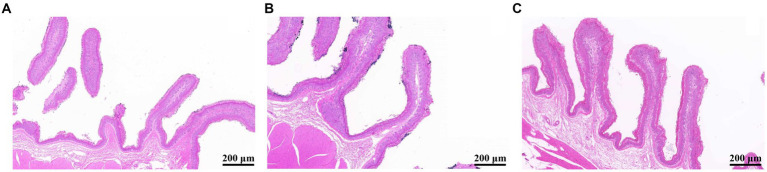
Effects of YC on the morphology of rumen epithelial tissue (200 μm). **(A)** CON, basal diet control; **(B)** LYC, 20 g/d YC added group; **(C)** HYC, 40 g/d YC added group.

No significant differences in final body weight, average daily gain, and average daily feed intake were observed, and the gain to feed ratio was higher in CON group than in HYC group, according to Supplementary Table S2. As shown in Figure 1, YC was generally able to alter rumen papillae development, and compared to the HYC group, the CON group had shorter papillae and signs of shedding. To further quantify the effects of YC on the rumen epithelium, we measured rumen epithelial parameters. The results are shown in Table 1, the rumen papilla length of the HYC group was significantly higher than that of the CON and LYC groups (*p* < 0.05). The stratum corneum thickness in control group was significantly greater than that of the other two groups (*p* < 0.05); YC did not have a significant effect on papilla width and rumen muscular thickness between the three groups (*p >* 0.05).

**Table 1:** Effects of YC on epithelial parameters of the rumen in sheep.

Item	CON	LYC	HYC	SEM	*P-*value
Ruminal papilla length (μm)	2415.2^b^	2414.2^b^	2706.9^a^	51.15	0.020
Ruminal papilla width (μm)	425.3	408.9	447.6	6.89	0.066
Stratum corneum thickness (μm)	71.8^a^	61.6^b^	65.8^b^	1.358	0.005
Muscle layer thickness (μm)	1652.9	1731.7	1781.3	29.4	0.203

^1a,b^Means within a same row followed by different lower case letters differ significantly among different groups (*p* < 0.05).CON, basal diet control; LYC, 20 g/d YC added group; HYC, 40 g/d YC added group, the same below.

### Effects of YC on the diversity of sheep rumen flora

**Figure 2:**
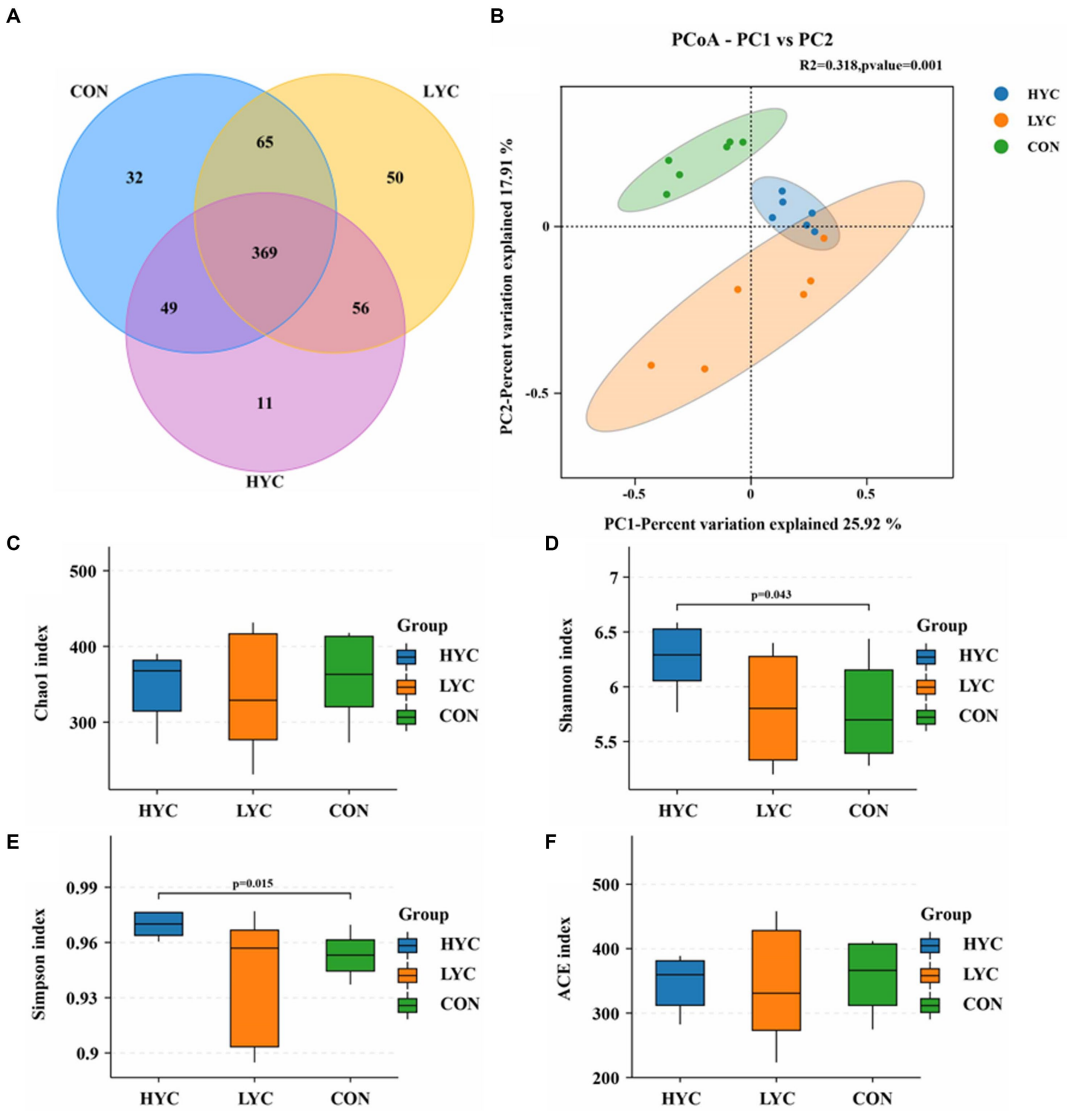
Effects of YC on rumen microbial diversity. **(A)** Venn diagram of rumen OUT. **(B)** PCoA analysis of rumen microorganisms. **(C)** Chao1 Index. **(D)** Shannon Index. **(E)** Simpson Index. **(F)** ACE Index.

The effects of YC on rumen microbial diversity are shown in Figure 2. A total of 632 valid OTUs were obtained from 18 samples of three groups after quality control. Venn diagrams demonstrated the number of OTUs shared by different groups (Figure 2A), confirming the effects of YC in sheep rumen microorganisms. The results of the alpha diversity analysis of rumen microbiota based on OTUs and species abundance were shown in Figures 2C–F. Shannon and Simpson indices in HYC group were significantly higher than other two groups (*p* < 0.05), and Chao1 and ACE indices were not significantly change. The results of PCoA based on Bray-curtis algorithm were shown in Figure 2B, the contribution of principal coordinate 1 (PCo1) and principal coordinate 2 (PCo2) were 25.92 and 17.91%, respectively, and the CON group was significantly distinguished from the two experimental groups (*p* < 0.05), indicating that YC addition affected the rumen flora structure of sheep to a greater extent. Moreover, the sample cluster analysis shows that the distance between the rumen microflora structure of the control group and test groups is significantly different, indicating that YC can change the rumen microflora structure of sheep.

### Effects of YC on the structure of rumen flora in sheep

At the phylum level, a total of 14 phyla were identified, and statistical analyses were performed for phyla with relative abundance percentages above 1%. The results are shown in Table 2, where Bacteroidetes, Firmicutes, Proteobacteria and Tenericutes accounted for more than 97% of the total and were the dominant phyla in the sheep rumen. Significance analysis showed that the Tenericutes in the HYC group was significantly higher compared to the other two groups (*p* < 0.05).

**Table 2:** Effects of YC on the relative abundance of rumen phylum in sheep rumen.

Phylum	CON	LYC	HYC	SEM	*P*-value
Bacteroidetes	44.99	41.01	47.29	2.435	0.352
Firmicutes	50.39	50.85	43.88	2.721	0.301
Proteobacteria	1.71	5.08	3.66	1.007	0.164
Tenericutes	1.60^b^	1.64^b^	3.37^a^	0.471	0.030

^1^CON, basal diet control; LYC, 20 g/d YC added group; HYC, 40 g/d YC added group.

A total of 143 genera were identified at the genus level, of which the 22 genera with a relative abundance of >1% were found in the three groups in the sheep rumen (Table 3). Compared with the CON group, the relative abundance of *Prevotella_1* was significantly decreased (*p* < 0.05), whereas the relative abundance of *Ruminococcaceae_UCG-005*, *uncultured_ bacterium_o_Mollicutes_RF39*, *Prevotellaceae_UCG-001*, *[Eubacterium]_nodatum_group*, *Desulfovibrio* and *Ruminococcus_1* were significantly increased in the LYC and HYC groups (*p* < 0.05). Furthermore, adding YC in the diet resulted in a trend of increasing the relative abundance of *Ruminococcus* and *Succiniclasticum* compared with the CON group (0.05 ≤ *p* < 0.1).

**Table 3:** Effects of YC on the relative abundance of bacteria at genus in sheep rumen.

Genus	CON	LYC	HYC	SEM	*P*-value
*Prevotella_7*	15.14	14.62	12.61	4.145	0.910
*Prevotella*	1.73	9.90	9.76	2.174	0.100
*Prevotella_1*	14.42^a^	3.74^b^	6.95^b^	2.281	0.025
*Uncultured_bacterium_f_Veillonellaceae*	5.54	3.53	6.08	1.314	0.442
*Rikenellaceae_RC9_gut_group*	6.73	4.37	6.05	1.347	0.531
*Ruminococcus*	5.63	2.98	6.04	0.844	0.052
*Ruminococcaceae_UCG-005*	0.85^b^	0.12^b^	4.08^a^	0.532	0.002
*Uncultured_bacterium_f_Muribaculaceae*	1.10	3.05	3.50	1.085	0.343
*Ruminococcaceae_UCG-014*	1.84	4.32	3.38	0.885	0.297
*Uncultured_bacterium_o_Mollicutes_RF39*	1.37^b^	1.57^b^	3.36^a^	0.485	0.021
*Prevotellaceae_UCG-001*	0.50	1.37	3.08	0.653	0.079
*Succiniclasticum*	11.14	11.09	2.87	2.052	0.057
*Dialister*	1.72	1.71	2.78	0.663	0.473
*[Eubacterium]_nodatum_group*	0.39^b^	1.28^a^	1.86^a^	0.248	0.010
*Desulfovibrio*	0.42^b^	1.24^a^	1.84^a^	0.268	0.023
*[Eubacterium]_ruminantium_group*	1.81	1.40	1.33	0.411	0.687
*Oribacterium*	0.63	0.91	1.28	0.311	0.410
*Uncultured_bacterium_f_Lachnospiraceae*	0.45^b^	1.30^a^	1.16^a^	0.183	0.030
*Christensenellaceae_R-7_group*	1.18	1.44	1.15	0.492	0.915
*Uncultured_bacterium_f_F082*	1.35	0.91	1.14	0.389	0.743
*U29-B03*	0.62	0.42	1.10	0.178	0.085
*Ruminococcus_1*	0.99^a^	0.35^b^	1.06^a^	0.189	0.036

^1^The table shows the genera with relative abundance > 1%.CON, basal diet control; LYC, 20 g/d YC added group; HYC, 40 g/d YC added group.

**Figure 3:**
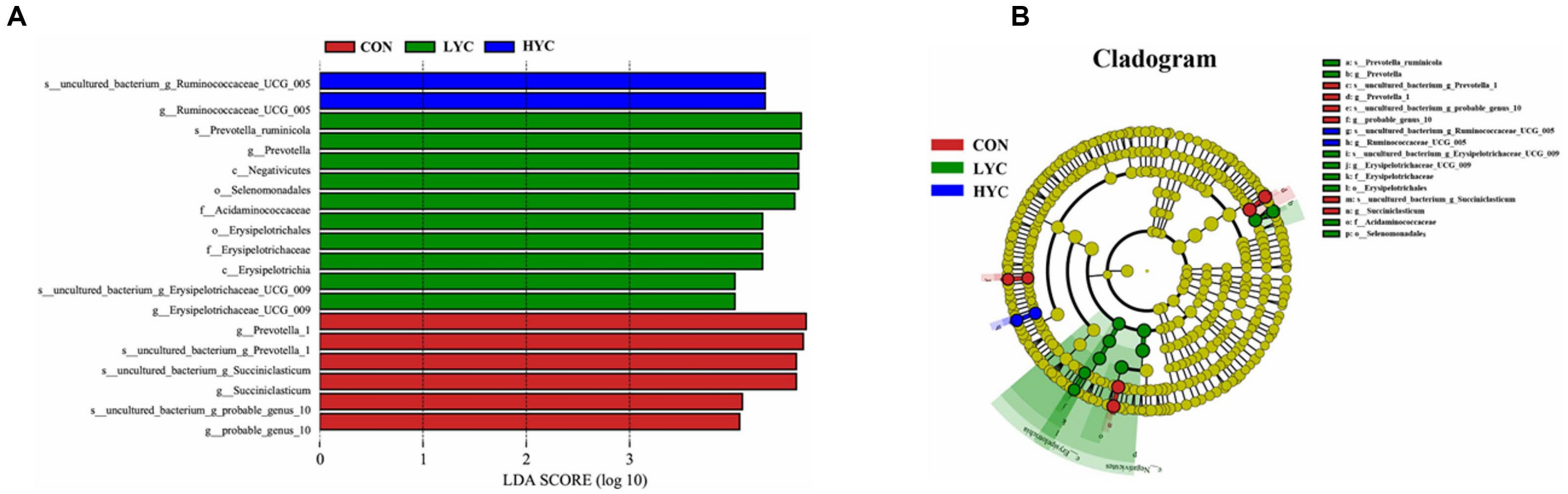
Microbial communities of different groups. **(A)** The LEfSe analysis histogram. Species with an LDA Score greater than a set value (set to 4.0 by default) are shown, with the length of the bars representing the effect size of the differing species (i.e., as an LDA logarithmic Score), and the different colors of the bar graph indicates the sample group corresponding to the taxon with higher abundance. **(B)** The LEfSe analysis branch diagram. The node size corresponds to the average relative abundance of the taxa. The yellow nodes represent taxa with insignificant differences between groups and nodes of different colors indicate microbiota that play an important role in the group represented by that color.

To further explore differences between samples, we performed LEfSe analyses (Figure 3). The more abundant bacterial genus in the CON group were *Prevotella1*, *probable_genus_10* and *Succiniclasticum*, the significantly abundant bacteria in the LYC group were *Prevotella_ruminicola, Prevotella, Erysipelotrichaceae_UCG_ 009* and others, whereas the significantly abundant bacteria in the HYC group were *Ruminococcaceae_UCG_005*. There were significant differences in the abundance of microbial communities among the CON group, LYC group and HYC group, indicating that dietary YC supplementation significantly improved the rumen microbial community structure.

### Effects of YC on the metabolite profiles of sheep

**Figure 4:**
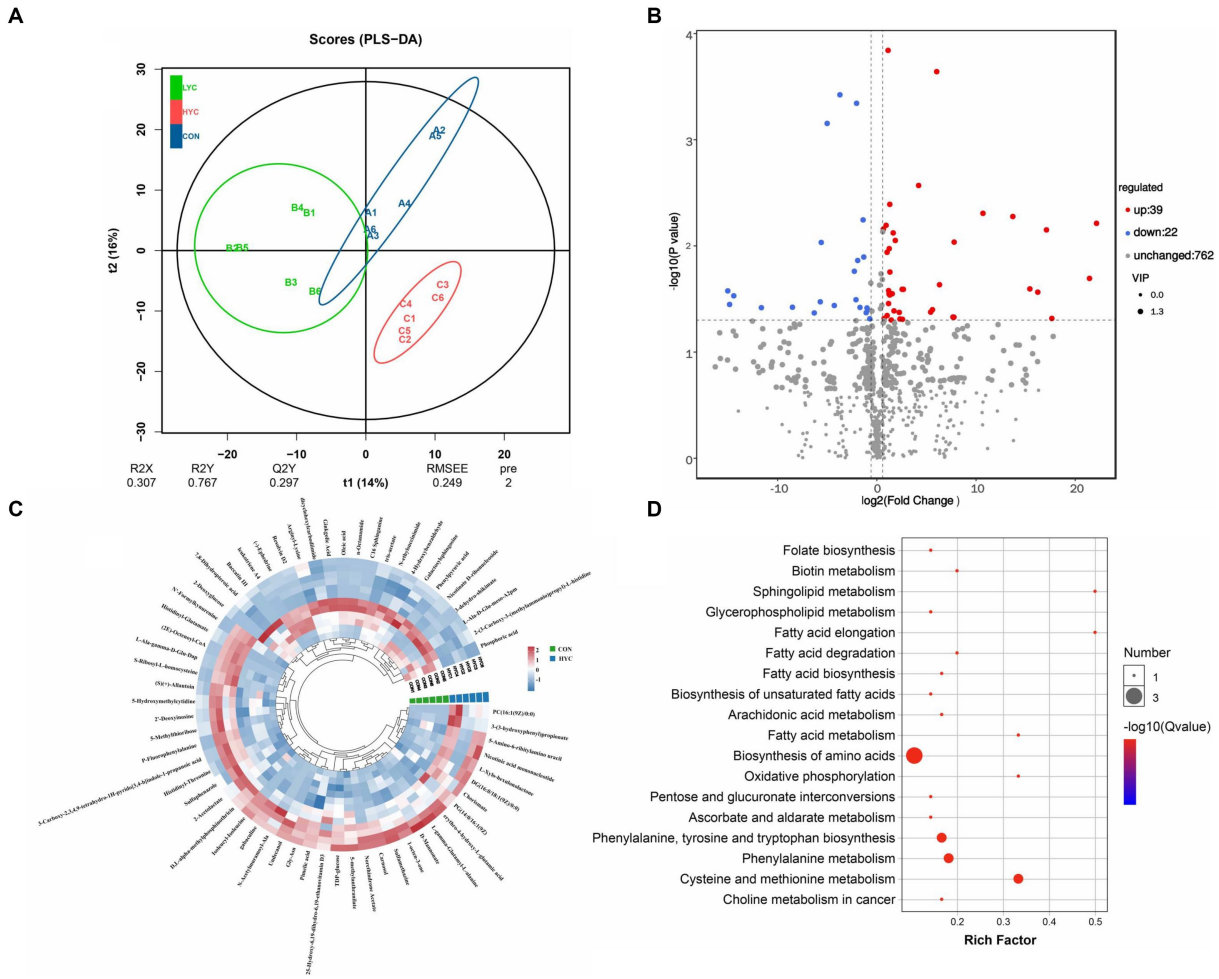
Effects of YC on rumen metabolites. **(A)** PLS-DA analysis of rumen metabolites. **(B)** Volcano plots of differential metabolites in CON vs. HYC groups. **(C)** Annular heat map of differential metabolites. **(D)** KEGG pathway enrichment bubble chart of differential metabolites in CON vs. HYC groups. CON, basal diet control; LYC, 20 g/d YC added group; HYC, 40 g/d YC added group.

**Figure 5:**
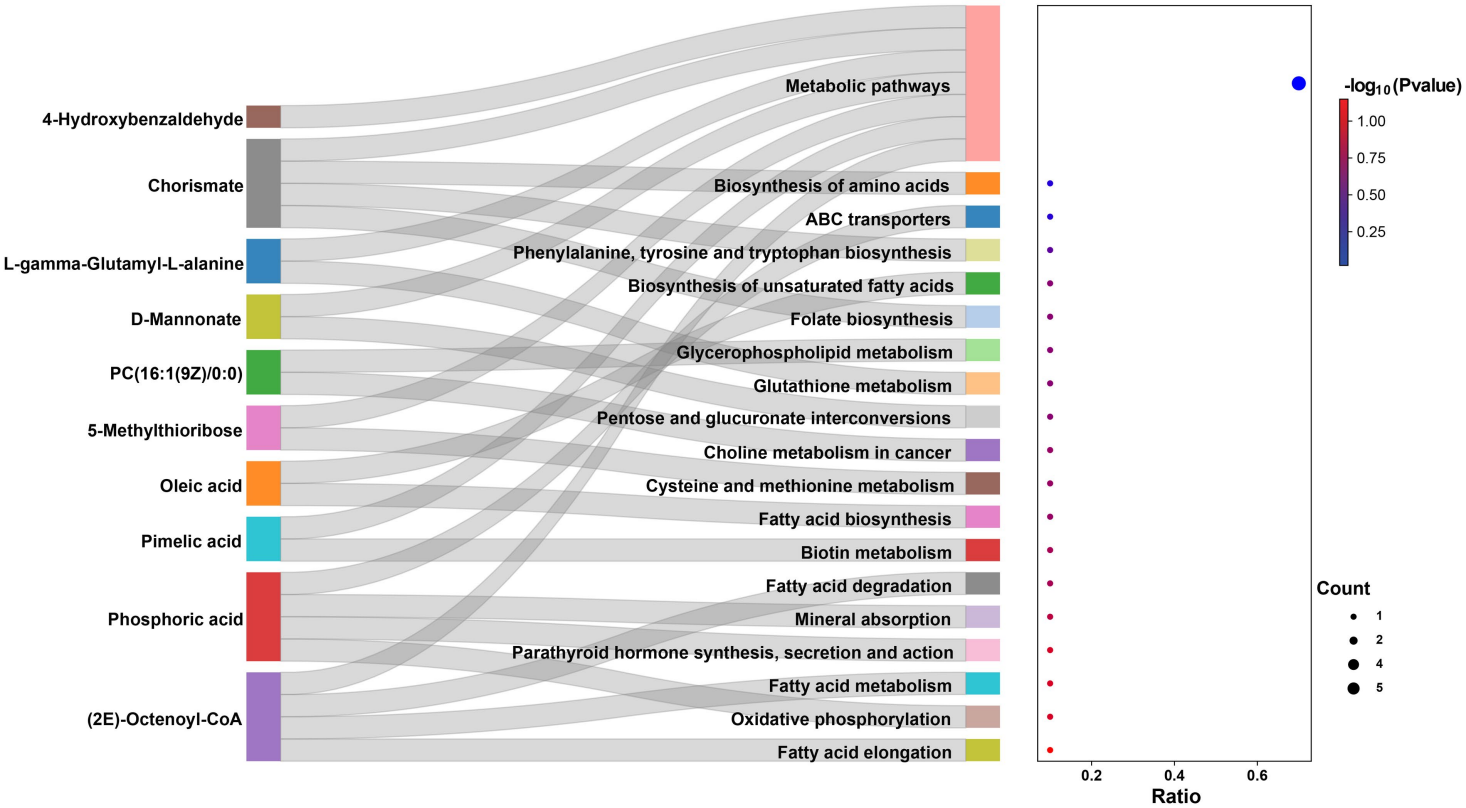
Sankey-Bubble Chart of the top 10 differential metabolites between CON vs. HYC.

A total of 823 metabolites were identified from all rumen fluid samples to assess the impact of YC on ruminal metabolite profiles in sheep. As shown in Figure 4A, the PLS-DA score plot showed good aggregation with significant differences among groups, and the random permutation test showed that the model derived from PLS-DA had good fit and high predictability (R2X = 0.653, R2Y = 0.991, Q2 = 0.92). Based on the PLS-DA results, it is evident that there was a significant separation of metabolites between the CON and HYC groups, with minimal intra-group variation. Thus, the differential metabolites between these two groups were screened and classified for subsequent analysis. Differential metabolites were screened by FC > 1.5, *p* < 0.05, and VIP > 1. A total of 61 differential metabolites were identified for CON vs. HYC with 39 up-regulated and 22 down-regulated metabolites (Figures 4B,C). Among them, these differential metabolites are mainly categorized as carboxylic acids and their derivatives, benzene and substituted derivatives, organic oxygen compounds and fatty acyl substances. To further explore the effects of YC in rumen metabolic profile in sheep, metabolic pathway enrichment analysis of differential metabolite was performed. The results were shown in Figure 4D, screened differential metabolites between CON and HYC groups were mainly enriched in several pathways related to nutritional metabolism, including fat digestion and absorption, glycerophospholipid metabolism and biosynthesis of unsaturated fatty acids. Subsequently, the top10 differential metabolites were annotated to specific metabolic pathways in the KEGG database, presented in Table 4 and Figure 5. The top 10 differential metabolites were Phosphoric acid, 5-Methylthioribose, Chorismate, Pimelic acid, PC(16:1(9Z)/0:0), L-gamma-Glutamyl-L-alanine, D-Mannonate, 4-Hydroxybenzaldehyde, (2E)-Octenoyl-CoA, and Oleic acid, which are mainly involved in amino acid metabolism, lipid metabolism and vitamin metabolism pathways. The vitamins in ruminants are synthesized by certain ruminal microbial taxa and are related to nutritional metabolism (such as fatty acid synthesis and gluconeogenesis). Notably, the results showed that most metabolites related to fatty acid metabolism and vitamin metabolism were upregulated, indicated a different requirement of vitamins for sheep rumen bacteria, and therefore an altered biological metabolism in activities such as fatty acid biosynthesis and Glycerophospholipid.

**Table 4:** Top10 differential metabolites that can be annotated to metabolic pathways.

Metabolite	VIP	regulated	KEGG_pathway_annotation
Phosphoric acid	2.27	Down	Mineral absorption, Oxidative phosphorylation; ABC transporters; Parathyroid hormone synthesis, secretion and action.
5-Methylthioribose	1.99	Up	Metabolic pathways; Cysteine and methionine metabolism.
Chorismate	1.93	Up	Phenylalanine, tyrosine and tryptophan biosynthesis; Biosynthesis of amino acids; Metabolic pathways; Folate biosynthesis.
Pimelic acid	1.86	Up	Biotin metabolism.
PC(16:1(9Z)/0:0)	1.80	Up	Glycerophospholipid metabolism; Choline metabolism in cancer.
L-gamma-Glutamyl-L-alanine	1.78	Up	Metabolic pathways; Glutathione metabolism.
D-Mannonate	1.77	Up	Pentose and glucuronate interconversions; Metabolic pathways.
4-Hydroxybenzaldehyde	1.76	Down	Metabolic pathways.
(2E)-Octenoyl-CoA	1.76	Up	Fatty acid degradation; Metabolic pathways; Fatty acid elongation; Fatty acid metabolism.
Oleic acid	1.76	Down	Fatty acid biosynthesis.

### Analysis of the association between rumen microorganisms and metabolites

**Figure 6:**
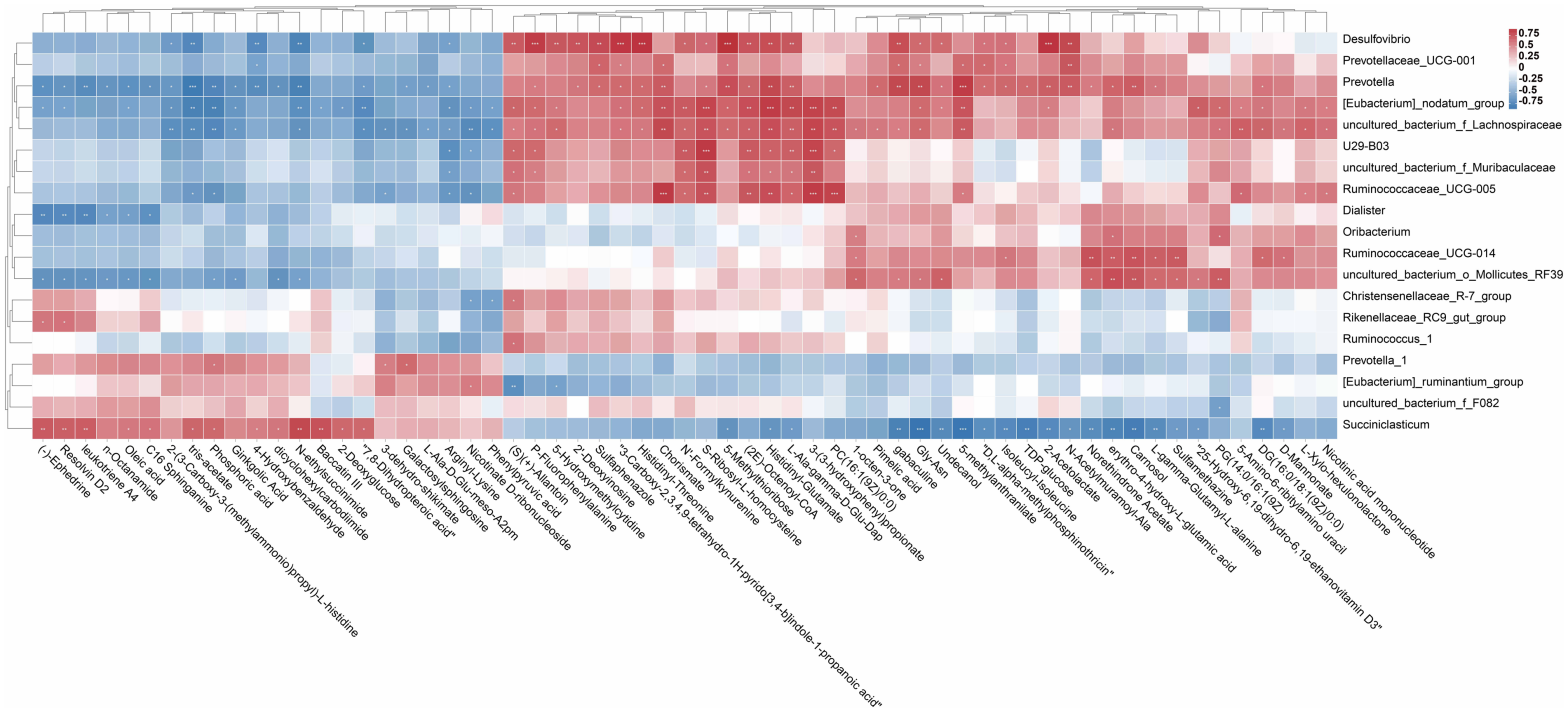
Rumen differential metabolites and microbial correlation heat map. Screening of rumen differential microorganisms with relative abundance >1% between CON vs. HYC. Data containing at least one set of correlation coefficients and correlation *p*-values consistent with |r| > 0.6 and *p* < 0.05 were retained and then plotted on a heat map. Asterisks indicate significant correlation between differential metabolites and microbial (*p < 0.05, **p < 0.01, and ***p ≤ 0.001).

To study the correlation among the changes in rumen microorganisms and metabolites, correlation analysis between significant differential metabolites and differential bacterial flora was performed by calculating Pearson’s correlation coefficient. As shown in Figure 6, these microorganisms (*uncultured_bacterium_o_Mollicutes_RF39*, *Ruminococcaceae_UCG-005*, *[Eubacterium]_nodatum_group*, *uncultured_bacterium_f_Lachnospiraceae*, and *Desulfovibrio*) significantly enriched in the HYC group (*p* < 0.05) displayed distinct correlations with metabolite categories, such as 4-Hydroxybenzalde Hyde, Phosphoric acid, Chorismate, D-Mannonate, L-gamma-Glutamyl-L-alanine, 5-Methylthioribose, PC(16:1(9Z)/0:0), (2E)-Octenoyl-CoA, and Pimelic acid, etc. The correlation between microbial abundance and metabolites revealed the relationship between rumen microbial groups and host metabolism.

**Figure 7:**
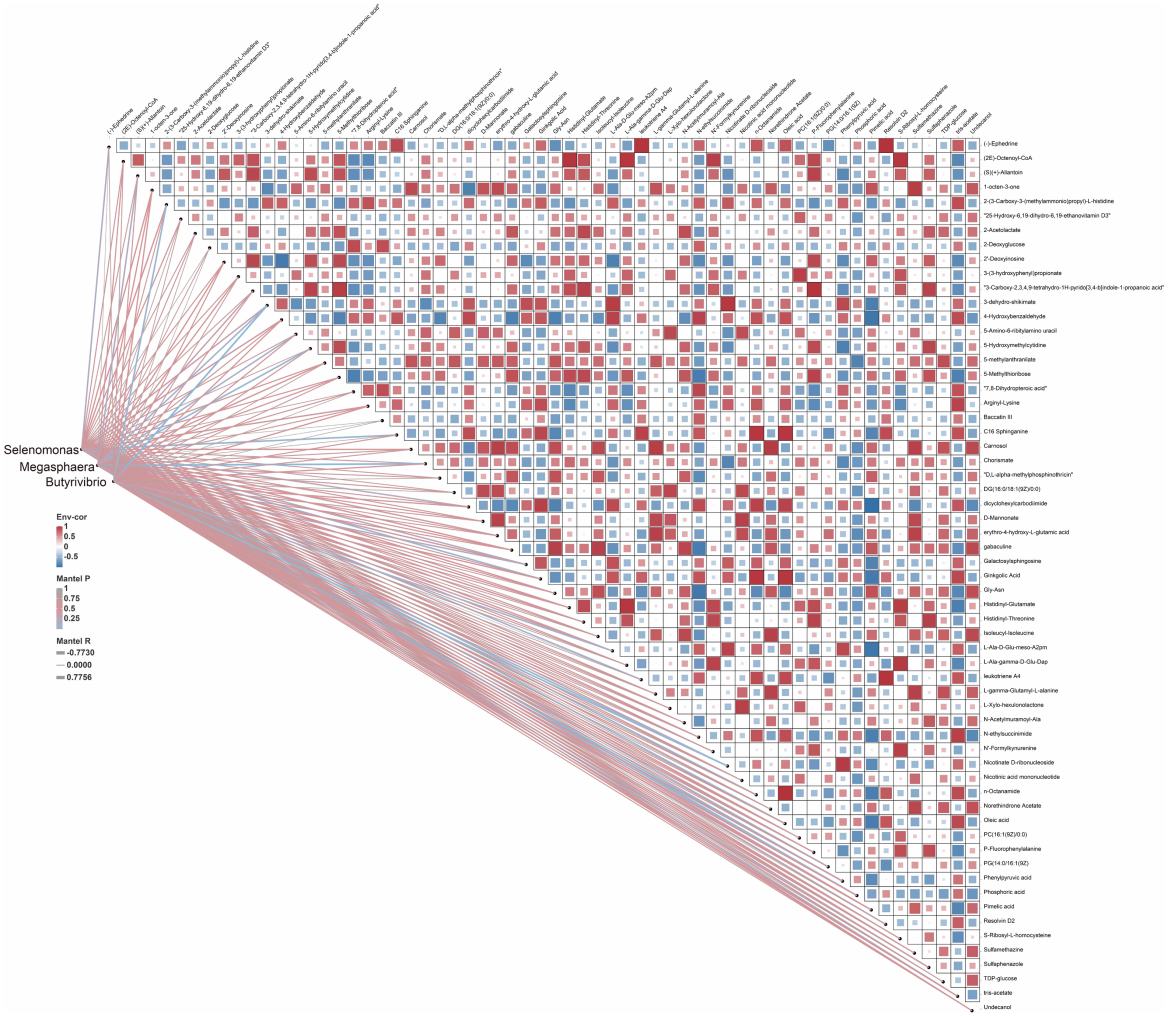
Heat map of differential metabolites and lactate metabolism-related microorganisms. Heat maps: the color of each matrix color block on the heat map represents the positive and negative correlation coefficient between metabolites, and the size of each matrix color block represents the absolute value of the correlation coefficient; network diagram: correlation of microbial abundance with each metabolite, and the line thickness and color indicates the correlation strength the significance degree, respectively.

Lactic acid accumulation is a major focus of ruminant research, and we subsequently further investigated the relationship between lactic acid-associated flora and rumen differential metabolites (Figure 7). The results of correlation analysis showed that *Selenomonas, Megasphaera* and *Butyrivibrio* showed significant positive correlation with Phosphoric acid, Oleic acid, 4-Hydroxybenzaldehyde and significant negative correlation with L-gamma-Glutamyl-L-alanine, (2E)-Octenoyl-CoA, D-Mannonate, 5-Methylthioribose, Chorismate, Pimelic acid.

## Discussion

Our previous research results (Su et al., 2022) showed that, adding YC to the diet can effectively improve the daily gain and feed-to-meat ratio of sheep, and increase the apparent digestibility of crude protein, NDF and ADF. Therefore, we speculated that this may be related to the effects of YC on the rumen epithelium. In the current study, YC could affect the rumen epithelium development, as seen from significantly increased rumen papilla length and reduced stratum corneum thickness. A well-developed rumen epithelium is the basis for ruminants to have a high absorption rate and barrier capacity. Lesmeister et al. (2004) believed that the rumen papilla height is the most important factor in evaluating the rumen development, followed by the papillae width and rumen wall thickness. The rumen papilla is a small protrusion of the rumen mucosal epithelium, which can increase rumen area for VFA absorption. Well-grown rumen papilla can ensure efficient absorption and utilization of nutrients, and well-developed rumen epithelium has many functions including nutrient absorption, metabolism, pH regulation, as well as immune and barrier functions, which are necessary conditions for maintaining rumen health in ruminants (Faniyi et al., 2019).

YC in the diet has positive effects on growth, rumen and intestinal tract in calves, improves rumen fermentation by increasing butyric acid production, and promotes the growth and development of rumen papilla, as reviewed by Alugongo et al. (2017). In addition, this positive effect is more pronounced in calves that have been stressed or exposed to different pathogenic factors (Alugongo et al., 2017). Interestingly, our previous study showed that YC increase butyrate and total volatile fatty acids concentration in sheep rumen fluid (Supplementary Table S3; Su et al., 2022), suggesting that YC might increase butyrate production by regulating rumen fermentation pattern, and hence repairing rumen epithelial injury, which is consistent with the results of our current study. High-concentrate diets have a negative impact on the rumen epithelium development and prolonged SARA can lead to atrophy of the rumen papilla, exacerbate the expansion of rumen trauma area, as well as finally affect rumen function and animal health.

A large number of studies have shown that YC promotes rumen development and improves rumen barrier function. Xiao et al. (2016) showed that 2% YC significantly increase the length, width and mucosal thickness of rumen papilla in calves. Although most studies have shown that YC can repair rumen epithelial injury and improve the ruminal papillae development, which is consistent with our results, there are a few studies that are different. For example, Magalhães et al. (2008) showed that YC have no significant effect on the length and width of rumen papilla and rumen wall thickness of calves aged 1 and 2 months. These inconsistent results may be related to the state of the animals themselves, with studies suggesting that YC plays a positive role when animals are stressed, sick or have nutritional imbalance. The improved development of the rumen by YC may be related to its regulation of rumen microflora, which affects the production of VFA, or it may also be protected by the ability of yeast polysaccharides to adsorb LPS and other endotoxins from the rumen, preventing them from entering the bloodstream via the rumen epithelium and causing inflammation, the exact mechanism of action of which needs to be studied in more depth.

Ruminal microorganisms can participate in nutrient digestion o by secreting digestive enzymes, which play an important role in regulating rumen fermentation and maintaining ruminal health (Fontanesi, 2016). Alpha diversity is assessed by a series of statistical indices to estimate the species abundance and diversity of environmental community. The ACE and Chao1 indices are mainly used to assess the number of OTUs in a sample, whereas the Shannon and Simpson indices mainly comprehensively reflect the diversity of species in a sample (Segata et al., 2011). Our results showed that YC significantly increased Shannon and Simpson indices in sheep rumen samples with a significant dose effect, which may imply that YC increases the species diversity of rumen microorganisms. It has been shown that higher rumen microbial diversity represents a more stable rumen microecosystem, which is important for ruminants to maintain health and improve production performance (Mccann et al., 2017). To further understand the effects of YC on rumen microorganisms in sheep, we analyzed the effect of YC on rumen flora structure at the phylum and genus level. The results showed that Bacteroidetes and Firmicutes were the two most dominant phylum in sheep rumen under high concentrate diets, and that YC significantly increased the relative abundance of Tenericutes. de Nardi et al. (2016) showed that a mixture additive of polyphenols and essential oils increased the abundance of many Tenericutes and decreased the abundance of species of Proteobacteria and Actinobacteria based on 16S rRNA detection techniques. At the genus level, we screened the genera with relative proportions >1% for analysis, and the results showed that YC significantly reduced the proportion of *Prevotella_1* and increased the proportions of *Ruminococcaceae_UCG-005*, *Prevotellaceae_UCG-001*, *Desulfovibrio*, and *Ruminococcus_1*. *Prevotella* spp. as one of the most abundant genera in the rumen can be involved in the degradation and digestion of several feed components. And two studies showed that the abundance of *Prevotella_1* was significantly higher in the high starch diets (Deaville and Galbraith, 1992; Michalak et al., 2021), which may imply a close relationship between this genus and starch degradation. The above speculations were also confirmed in a follow up study showing that *Prevotella ruminicola* is an important starch-and protein-degrading bacterium and that YC can reduce the abundance of rumen starch-utilizing bacteria and maintain rumen pH at healthy levels (Halfen et al., 2021), which is consistent with our results. Yeast (*S. cerevisiae*) fosters and stimulates the proliferation of lactic acid-utilizing bacteria (LUB), especially promoting the growth of *M. elsdinii populations* (Rossi et al., 2004; Sirisan et al., 2013). This effect is attributed to the high concentration of growth factors derived from the yeast cell walls (Rossi et al., 2004). Furthermore, *S. bovis* and *S. cerevisiae* are both capable of competing for glucose via growth promoters (Rossi et al., 2004; Sirisan et al., 2013). Ruminococcaceae, as an important member of the Firmicutes, can help ruminants utilize crude fiber for fermentation and produce large amounts of VFA (Liu et al., 2020). A large number of studies have shown that *S. cerevisiae* products, either ADY or YC, can significantly increase the abundance of *Ruminococcus* by increasing the abundance of cellulolytic bacteria, lactic acid-utilizing bacteria, and carbohydrate-active enzymes in the rumen (Yohe et al., 2015; Ogunade et al., 2019). These results are consistent with our study showing that YC increased the relative abundance of *Ruminococcaceae_UCG-005* and *Ruminococcus_1*. Interestingly, our previous study using metagenomic technology also concluded that YC can increase the abundance of *R. flavefaciens* and *Ruminococcus* (Su et al., 2022). These results suggested that YC can alter the abundance of cellulose-degrading bacteria in the sheep rumen. Changes in the microbial flora can degrade rumen cellulose and hemicellulose to produce VFA to provide energy for the body and promote rumen epithelium development. Studies have shown that *Desulfovibrio* is a sulfate-reducing bacterium in the ruminant’s rumen, and the sulfide produced by the sulfate reduction process can hinder the oxidation of short-chain fatty acids in the rumen, and it may be related to the low nitrogen utilization efficiency of dairy cows (Jia et al., 2012; Xue et al., 2020). In our study, YC increased the abundance of *Desulfovibrio* but without negatively affecting the animals, which might be related to diet type and animal breed for reasons that are not yet clear.

The above results indicated that YC affected the development of rumen papillae and rumen flora. To further explore the effect of adding YC on rumen metabolites, we analyzed the metabolic changes of rumen metabolites using a untargeted LC–MS-based metabolomics approach. Our research shows that adding YC has a greater impact on rumen metabolites, which mainly belong to amino acids and their metabolites, organic acids and their derivatives, carbohydrates and their metabolites, and glycerophospholipids. Studies have shown that the main metabolites in the rumen, such as organic acids, amino acids and sugars, are mainly produced by rumen microorganisms digesting the diet (Scano et al., 2014). This might imply that YC modulates the rumen metabolite profile mainly through alterations in rumen microorganisms, which ultimately have an effect on the host animal. Pathway enrichment analysis of these metabolites revealed that the differential metabolites were mainly focused on fatty acid metabolism-related pathways, mainly including fat digestion and absorption, glycerophospholipid metabolism and unsaturated fatty acid biosynthesis. Ding et al. (2014) showed that although high concentrate can improve the body’s fat metabolism, it will damage the integrity of the inner wall of the animal’s gastrointestinal tract, whereas yeast can improve fat and phospholipid metabolism, which is conducive to maintaining the integrity of the inner wall cells of the digestive tract. Adding YC to the diet may promote the hydrogenation of rumen microorganisms, and it has been shown that adding YC to high-fat diets can increase the concentration of saturated fatty acids in sheep rumen (Lascano et al., 2012). These results are consistent with our results that YC mainly affected rumen fatty acid metabolism. It should be noted that there are also differences in metabolites related to vitamin metabolic pathways such as biotin and folic acid. Based on this, we speculated that YC might have a potential effect on B vitamin metabolism in ruminants. Many studies have shown that thiamin, a B vitamin, alleviate a series of adverse effects caused by SARA in animals by increasing the abundance of rumen catabolic bacteria to enhance the expression of rumen epithelial tight junction proteins, and reducing the secretion of inflammatory factors (Pan et al., 2017; Ma et al., 2021). It has also been shown that biotin and B-complex vitamins alleviate rumen acidosis in ruminants and are effective in the prevention and treatment of hoof disease in dairy cows (Bergsten et al., 2003; Bampidis et al., 2007). These studies demonstrate the importance of B vitamins for ruminant health, especially when animals are in a high concentrate feeding mode. It is well known that YC cultures contain many nutrients, including B vitamins. In this study, YC affected metabolites associated with the B vitamin metabolic pathway, and whether this alteration is due to the B vitamins it contains or through changes in the rumen flora associated with B vitamin synthesis is still unclear. Interpretation of these issues were important for the use of YC to meet the B vitamin requirements of ruminants, and needs to be focused on in future studies. Notably, the sources of vitamin B are mainly from the synthesis of rumen microorganisms. Only by clarifying the relationship between microorganisms and B vitamin synthesis can the synthesis of B vitamins be regulated through animal nutrition, so as to better maintain ruminant health and improve production performance.

## Conclusion

In summary, YC might improve rumen barrier function by repairing rumen epithelial injury, such as repairing rumen papilla length and stratum corneum thickness. Furthermore, YC can significantly improve the rumen flora of sheep fed with high-concentrate conditions, increase the relative abundance and diversity of rumen microorganisms, reduce the flora associated with starch degradation, and increase the abundance of flora associated with fiber decomposition. Changes in the microbiota further affected the rumen metabolic profile, resulting in differences in metabolites related to amino acid metabolism, lipid metabolism, and vitamin metabolism. These variations might be what led to the change in rumen fermentation type in our previous study and maintained a healthy rumen under high concentrate conditions. The above results indicated that an addition of 40 g/d YC to the diet was helpful to repair rumen epithelial injury under high concentrate conditions, thereby maintaining rumen health in sheep.

## Data availability statement

The datasets presented in this study can be found in online repositories. The names of the repository/repositories and accession number(s) can be found at: NCBI—PRJNA1023752.

## Ethics statement

The animal studies were approved by the Animal Care Committee of Gansu Agricultural University (GSAU-AEW-2020-0057). The studies were conducted in accordance with the local legislation and institutional requirements. Written informed consent was obtained from the owners for the participation of their animals in this study.

## Author contributions

HW: Conceptualization, Methodology, Software, Visualization, Writing – original draft. MS: Conceptualization, Methodology, Software, Writing – review & editing. CW: Formal analysis, Software, Writing – review & editing. DL: Formal analysis, Software, Writing – review & editing. QL: Formal analysis, Writing – review & editing. ZL: Formal analysis, Writing – review & editing. XQ: Formal analysis, Writing – review & editing. YW: Formal analysis, Writing – review & editing. YZ: Conceptualization, Supervision, Writing – review & editing. TL: Conceptualization, Methodology, Software, Writing – review & editing. YM: Conceptualization, Funding acquisition, Project administration, Supervision, Writing – review & editing.

## Footnotes

^1^http://www.biocloud.net/

^2^https://qiime2.org/

^3^http://huttenhower.sph.harvard.edu/lefse/
