# Supplementary material for: Yeast culture repairs rumen epithelial injury by regulating microbial communities and metabolites in sheep
Source: Front Microbiol. 2023 Dec 1;14:1305772. doi: 10.3389/fmicb.2023.1305772 (PMC10722269; doi:10.3389/fmicb.2023.1305772)
Supplement: Supplementary file 1 [file Table_1.DOCX]

Supplementary Material

# Supplementary Figures and Tables

## Supplementary Tables

**Supplementary Table 1.** The ingredients and nutrient composition of the basal diet (DM basis)**.**

| **Item** | **Diet** |
| --- | --- |
| **Ingredients, (% of DM)** |  |
| Ground Corn | 35.4 |
| Wheat bran | 10.5 |
| Soybean Meal | 10.8 |
| Rapeseed Meal | 4.3 |
| Cottonseed meal | 4.4 |
| Oat hay | 14 |
| Alfalfa hay | 13.3 |
| Corn silage | 4.1 |
| Limestone | 1.2 |
| Salt | 1.0 |
| Vitamin-mineral mix^1^ | 1.00 |
| **Nutrition composition^2^** |  |
| DM, % | 82.65 |
| Crude protein, %DM | 16.17 |
| Crude fat, %DM | 2.74 |
| NDF, %DM | 29.6 |
| ADF, %DM | 17.09 |
| Ca, %DM | 0.67 |
| P, %DM | 0.41 |

DM, dry matter; NDF, neutral detergent fiber; ADF, acid detergent fiber.

^1)^ The premix provided the following per kg of diets: Fe 430 mg, Zn 950 mg, Cu 650 mg, I 45 mg, Mn 600 mg, Se 30 mg, Co 20 mg, VA 120,000 IU，VE 8000 IU, VD 40,000 IU, and 2300 IU of VK.

^2)^ Measured values. Main instruments and equipment: Electric constant temperature drying oven DHG-9070A (Marit Technology Co., LTD, China), Kjeldahl nitrogen analyzer 8400 (Foss, Sweden), Ceramic Fiber Muffle Furnace SX2-16-10A (Marit Technology Co., LTD, China), visible spectrophotometer V-1500 (Macylab instrument, China).

**Supplementary Table 2.** Effects of YC on growth performance of sheep.

| **Item** | **CON** | **LYC** | **HYC** | ***P-*value** |
| --- | --- | --- | --- | --- |
| Final body weight (kg) | 37.48±2.51 | 39.02±2.93 | 39.95±2.55 | 0.245 |
| Average daily gain (g·d^－1^) | 276.95±18.83 | 287.31±29.05 | 293.73±36.83 | 0.557 |
| Average daily feed intake (g·d^－1^) | 1189.71±392.61 | 1162.29±349.18 | 1106.77±382.26 | 0.226 |
| F/G | 4.31±0.29 | 4.08±0.41 | 3.82±0.50 | 0.094 |

^1)^ a, b Means within a same row followed by different lower case letters differ significantly among different groups (*P* < 0.05). CON: basal diet control; LYC: 20 g/d YC added group; HYC: 40 g/d YC added group. Feed to gain ratio (F/G).

**Supplementary Table 3.** Effects of YC on fermentation parameters of the rumen in sheep.

| **Item** | **CON** | **LYC** | **HYC** | **SEM** | ***P-*value** |
| --- | --- | --- | --- | --- | --- |
| pH | 6.35 | 6.45 | 6.49 | 0.04 | 0.182 |
| Acetate, mmol/L | 50.11 | 49.16 | 57.16 | 3.04 | 0.086 |
| Propionate, mmol/L | 19.53 | 22.04 | 23.00 | 1.55 | 0.065 |
| Butytate, mmol/L | 15.66^b^ | 16.32^b^ | 19.65^a^ | 0.73 | <0.01 |
| TVFA, mmol/L | 85.30^b^ | 87.51^b^ | 99.81^a^ | 3.20 | 0.020 |

^1)^ a, b Means within a same row followed by different lower case letters differ significantly among different groups (*P* < 0.05). CON: basal diet control; LYC: 20 g/d YC added group; HYC: 40 g/d YC added group. TVFAs: total volatile fatty acids.
